# Physiological mechanisms linking cold acclimation and the poleward distribution limit of a range-extending marine fish

**DOI:** 10.1093/conphys/coaa045

**Published:** 2020-09-08

**Authors:** Barrett W Wolfe, Quinn P Fitzgibbon, Jayson M Semmens, Sean R Tracey, Gretta T Pecl

**Affiliations:** Institute for Marine and Antarctic Studies, University of Tasmania, Hobart, Tasmania 7001, Australia

**Keywords:** Chrysophrys auratus, swim tunnel respirometry, thermal biology, range extension, aerobic scope, Tasmania, Australia

## Abstract

Extensions of species’ geographical distributions, or range extensions, are among the primary ecological responses to climate change in the oceans. Considerable variation across the rates at which species’ ranges change with temperature hinders our ability to forecast range extensions based on climate data alone. To better manage the consequences of ongoing and future range extensions for global marine biodiversity, more information is needed on the biological mechanisms that link temperatures to range limits. This is especially important at understudied, low relative temperatures relevant to poleward range extensions, which appear to outpace warm range edge contractions four times over. Here, we capitalized on the ongoing range extension of a teleost predator, the Australasian snapper *Chrysophrys auratus*, to examine multiple measures of ecologically relevant physiological performance at the population’s poleward range extension front. Swim tunnel respirometry was used to determine how mid-range and poleward range edge winter acclimation temperatures affect metabolic rate, aerobic scope, swimming performance and efficiency and recovery from exercise. Relative to ‘optimal’ mid-range temperature acclimation, subsequent range edge minimum temperature acclimation resulted in absolute aerobic scope decreasing while factorial aerobic scope increased; efficiency of swimming increased while maximum sustainable swimming speed decreased; and recovery from exercise required a longer duration despite lower oxygen payback. Cold-acclimated swimming faster than 0.9 body lengths sec^−1^ required a greater proportion of aerobic scope despite decreased cost of transport. Reduced aerobic scope did not account for declines in recovery and lower maximum sustainable swimming speed. These results suggest that while performances decline at range edge minimum temperatures, cold-acclimated snapper are optimized for energy savings and range edge limitation may arise from suboptimal temperature exposure throughout the year rather than acute minimum temperature exposure. We propose incorporating performance data with *in situ* behaviour and environmental data in bioenergetic models to better understand how thermal tolerance determines range limits.

## Introduction

Among the most pervasive consequences of climate change are species redistributions. Globally, species geographic range limits are shifting in latitude, elevation or depth, typically in the direction of the movement of local climate conditions (Chen *et al.*, 2011; Brito-Morales *et al.*, 2018). Marine range shifts are occurring at an average rate of 72 km per decade, over four times faster than those on land (Sorte *et al.*, 2010; Chen *et al.*, 2011; Poloczanska *et al.*, 2013) and have affected commercially important fisheries targets, altered distributions of critical habitat-forming species and led to the formation of novel ecological communities (Pecl *et al.*, 2017). Thus, this phenomenon has critical implications for biodiversity and conservation. Improving our understanding and capacity to forecast ongoing and future marine species redistributions remains a key challenge.

Much of the efforts to date towards understanding effects of climate on marine species and ecosystems, and especially marine species redistributions, have used statistical approaches, correlating historical ecological data (e.g. distribution, abundance or phenology) to environmental data to identify the factors driving observed trends (Perry *et al.*, 2005; Sunday *et al.*, 2012; Brodie *et al.*, 2015; Hill *et al.*, 2015; Pacifici *et al.*, 2015). These statistical relationships can be extrapolated with forecasted environmental conditions to predict future species distributions (García Molinos *et al.*, 2015; Champion *et al.*, 2019). The statistical approach often has the benefit of drawing upon large numbers of existing historical species records, which has been successful at identifying global scale patterns in range shifts. For example, 80% of range shifts occur in the same geographical direction as mean temperature change (Poloczanska *et al.*, 2013). However, there is substantial variation in the rate and direction of species redistributions not explained by climate alone that depends at least partially on species-specific biology (Sunday *et al.*, 2015).

A mechanistic approach towards understanding population responses to climate change is a complementary alternative to the statistical approach. When using a mechanistic approach, researchers aim to determine how environmental factors affect key biological processes, which can then be used to predict outcomes at a population level (Gibert *et al.*, 2015; Pacifici *et al.*, 2015). Mechanistic modelling approaches have the potential to make actionable predictions based on potential environmental perturbations (Jørgensen *et al.*, 2016). Typically, the relationships between environmental conditions and biological processes are quantified through empirical experiments focused on a fitness-linked performance at anywhere from the molecular to the ecological level (e.g. enzyme activity to prey attack rate) but often at the whole organism level. A substantial body of this research has been conducted to understand climate change impacts, often intuitively concentrated on response to high temperature for species of interest (Dell *et al.*, 2011; Seebacher *et al.*, 2014) and critical (i.e. lethal) temperature limits (Peck *et al.*, 2009).

The rate of leading range edge extensions at the cooler range limit of marine species is outpacing the contraction of warmer trailing range edges by over four times on average (Poloczanska *et al.*, 2013). As a result of species entering areas faster than leaving them in all but the tropical latitudes, range extensions are projected to be a primary driver of future biodiversity change in sub-tropical and temperate marine ecosystems (García Molinos *et al.*, 2015; Pecl *et al.*, 2017). Given the direction of range extensions is concurrent with the velocity of climate change (Poloczanska *et al.*, 2013), range extensions and in turn much of regional-level biodiversity changes are likely moderated by low, not high temperature tolerance. Despite the critical implications of cold tolerance on future biodiversity change, there is a paucity of experimental work on the topic. Moreover, many marine species do not encounter temperatures approaching lethal levels at range edges (e.g. Mora and Ospína, 2001; Eme and Bennett, 2009) and mobile species often have the behavioural capacity to avoid unfavourable temperatures (Mazaris *et al.*, 2009; Breau *et al.*, 2011; Whitlock *et al.*, 2015). If the thermal component of range limitation is determined by non-critical temperature effects, they are more relevant than lethal extremes for understanding range extensions. To resolve climate-driven global trends in marine biodiversity, more work is needed to understand how ecologically relevant cold temperatures influence fitness-linked performance and ultimately range limits.

Effectively employing a mechanistic approach towards species redistribution will require identifying and prioritizing the study of key measurable processes. While virtually all physiological processes in ectotherms are thermally sensitive, it would be inefficient if not impossible to experimentally measure all of them for each species of interest. Key processes should explain fitness limitation at ecologically relevant temperatures, such as at edges of ongoing range shifts and ideally be applicable across a range of species. Prospective key processes should link to high-level, ecologically relevant performances and behaviours (e.g. foraging, predator escape) and should be considered in the context of the life history and behavioural trade-offs with which processes have co-evolved, e.g. energy budgeting conflicts, foraging rate increasing growth but also predation risk (Holt and Jørgensen, 2015; Chuang and Peterson, 2016; Jørgensen *et al.*, 2016; Lancaster *et al.*, 2017; Biro *et al.*, 2018).

Aerobic scope, an organism’s oxygen transport capacity available to support any processes beyond maintaining homeostasis (e.g. growth, digestion, reproduction), has been suggested as the overarching physiological determiner of thermal limits (Pörtner and Farrell, 2008). This oxygen- and capacity-limited thermal tolerance (OCLTT) hypothesis proposes that as aerobic scope supports fitness-linked performance, an organism should have evolved such that fitness is maximized at the temperature where aerobic scope is optimized. With increasing distance above or below this proposed optimal temperature, aerobic scope should decrease until it becomes fitness-limiting towards the edges of the thermal window due to insufficient oxygen transport capacity (Pörtner, 2001; Pörtner and Knust, 2007). If aerobic scope drives thermal limitation consistently across a range of ectothermic species, the OCLTT would provide a unifying concept for how thermal stressors affect organisms and their biogeography (Portner and Peck, 2010), facilitating our ability to mechanistically model range shifts for many species efficiently (Cucco *et al.*, 2012).

Payne *et al.* (2016) demonstrated the relationship between aerobic scope’s optimal and upper critical (i.e. where aerobic scope declines to zero) temperatures closely corresponds to the relationship between *in situ* performance-optimizing temperature and warm range limit maximum temperatures across fish taxa. This suggests thermal aerobic scope limitation may explain range limits but was not tested at cold range limit temperatures. The OCLTT remains contentious; however, as for some species, temperatures that maximize aerobic scope have little relevance to real-world performance or temperature preference (Fry, 1947; Clark *et al.*, 2013; Norin *et al.*, 2014; Jutfelt *et al.*, 2018). The hypothesis predicts different mechanisms of oxygen limitation at low versus high temperatures (Verberk *et al.*, 2016; MacMillan, 2019) and thus could have predictive power at either temperature extreme even if not relevant at temperature optima or the other extreme. For example, energy allocation modelling of cod (*Gadus morhua*) based on the OCLTT predicts that fitness is optimized at a considerably lower temperature than aerobic scope, yet at temperature extremes, aerobic scope budgeting conflicts limit performance (Holt and Jørgensen, 2015). The OCLTT has scarcely been tested at long-term, sub-lethal temperatures (Verberk*, et al.*, 2016) or under naturally varying conditions (Morash *et al.*, 2018) relevant to range extensions. Thus, tests of this framework in the context of species’ range extensions are important because if the OCLTT has limited power to predict species’ cold range limits, research effort can be more fruitfully allocated towards other potential mechanisms (Clark *et al.*, 2013; Sinclair *et al.*, 2016; MacMillan, 2019).

Ocean warming hotspots, regions of the oceans undergoing rapid climate change, are useful natural laboratories to study various biological responses to warming (Hobday and Pecl, 2014). Use of these fast-warming regions might be well-placed to address questions relating to the specific physiological mechanisms underlying climate-driven species redistributions. Studying individuals occurring at contemporary range extension fronts allows for observations to reflect *in situ* intraspecific trait variation along with the often considerable physiological effects of natural thermal variation (Morash *et al.*, 2018). Intraspecific physiological and behavioural responses to temperature can vary across a population’s range due to acclimation, phenotypic plasticity and selection pressure at range edges (Donelson *et al.*, 2012; Donelson *et al.*, 2019). As individuals in newly extended range areas are likely to vary in fitness-related traits (Myles-Gonzalez *et al.*, 2015), experiments using individuals from elsewhere in the range may not reflect dynamics at the range extension front.

Southeast Australia is an ocean warming hotspot (Pecl *et al.*, 2014), with temperatures rising at a rate almost four times the global average due to a climate-driven extension of the East Australian Current (Cai *et al.*, 2005). Poleward species range shifts and out-of-range occurrences have been documented for dozens of taxa (Robinson *et al.*, 2015; Sunday *et al.*, 2015; Pecl *et al.*, 2019). The Australasian snapper *Chrysophrys auratus* (herein ‘snapper’) is a long-lived teleost fish (family Sparidae) currently undergoing a range extension into southeast Tasmania and provides an opportunity to quantify thermal sensitivity of performance at range-extension-front conditions.

To determine the degree to which different individual-level performances of this marine predator may relate to range limitation, we measured: (i) allometric scaling of metabolic rate, (ii) aerobic scope, (iii) swimming efficiency, (iv) performance and (v) recovery after exhaustive exercise using swim tunnel respirometry. We compared these performances in snapper collected from their most poleward known distributional extent after acclimation to a mid-range 20°C and after subsequent reacclimation to ambient range edge winter (minimum) temperatures (10–12°C) to investigate three hypotheses. Firstly, that aerobic scope should decline at range edge minimum temperature due to physiological tolerance-biogeography coupling (Pörtner, 2001; Portner and Peck, 2010; Payne *et al.*, 2016). Second, if oxygen transport capacity is the organismal-level determiner of cold performance limitation at ecologically relevant conditions (Pörtner, 2002; Pörtner and Knust, 2007), then we predict range edge cold acclimation will reduce snapper swimming and recovery performance commensurate with aerobic scope. Finally, if aerobic scope budgeting conflicts are a mechanism of cold-acclimated fitness limitation, we predict swimming and recovery will utilize a greater proportion of aerobic scope after range edge cold acclimation (Holt and Jørgensen, 2015).

## Methods

### Snapper collection and acclimation

**Figure 1:**
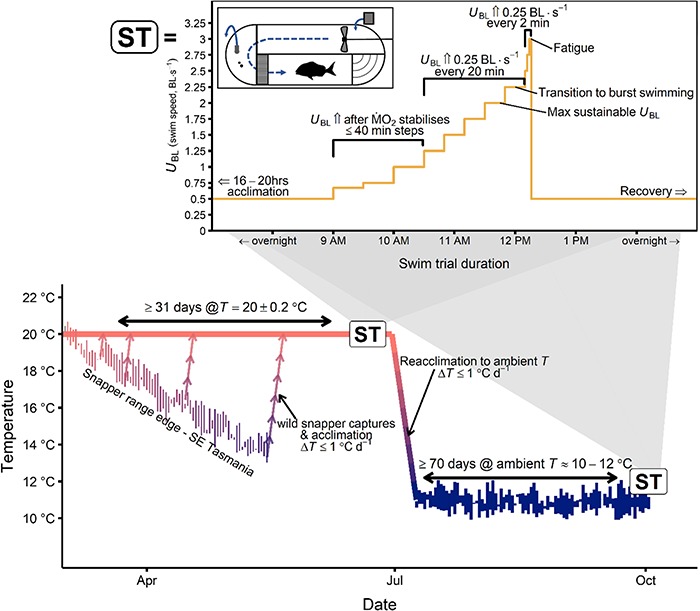
Snapper temperature acclimation schedule and swim trial experimental design (ST; inset). Beginning around the peak of seasonal water temperature, 14 wild snapper were captured on four occasions in southeast Tasmania at the poleward-most known extent of the population’s range. They were then acclimated at 20°C in a temperature controlled tank. After swim tunnel trials (ST), fish were reacclimated to the then winter ambient water temperature and transferred to a flow-through ambient tank, where the mean hourly temperatue across the acclimation period was 10.9 ± 0.4°C. Swim trials were repeated at 12°C and tank temperature at the time of transfer to the swim tunnel respirometer was within one degree of 12°C. *Inset:* swim trials began with overnight acclimation to the swim tunnel respirometry, at a swimming speed (*U*_BL_) that induced a resting gait (generally 0.5 body lengths [BL] s^-1^). For the first three incremental *U*_BL_ steps at ⅔, ¾ and 1 BL s^-1^ the fish was allowed to acclimate to the speed increase for up to 40 min or until oxygen consumption measurements (*Ṁ*_O2_) stabilized. Subsequently, *U*_BL_ was increased every 20 min by ¼ BL s^-1^ until the fish could no longer sustain the speed with a regular steady gait. At the completion of the *U*_BL_ step where gait transition occured, a constant acceleration trial was initiated where *U*_BL_ was increased by ¼ BL s^-1^ every 2 min until the fish was exhausted (made contact with the rear of the working section twice in a 30 s period), at which point *U*_BL_ was returned to rest and the fish was allowed to recover overnight. For more details of the swim tunnel respirometer, see Fig. 2.

Fourteen snapper (*Chrysophrys auratus*, 21.7–49.5 cm total length) were caught by hook and line in greater Storm Bay, southeast Tasmania (43.0°S, 147.7°E). This is the poleward-most known extent of the species’ Australian population. Snapper were captured on four occasions between March–May 2017 (Fig. 1). Ambient surface water temperature at the location and time of capture ranged from 19.7°C to 12.7°C. Snapper were transferred to the Institute for Marine and Antarctic Studies aquaculture facility the day of capture, double-tagged with Hallprint T-Bar Anchor tags for individual identification and housed in 4000 L holding tanks within ~1°C of ambient temperature at capture.

Snapper were acclimated to 20 ± 0.2°C at a rate of 1°C day^−1^. This 20°C is the maximum sea surface temperature reached in greater Storm Bay and was selected as an ‘optimal’ mid-range experimental temperature as it maximized wild juvenile snapper growth rates and adult reproductive growth. This 20°C is also the mean long term annual sea surface temperature at the latitude of maximal growth rates (Reynolds *et al.*, 2002; Murphy *et al.*, 2013; Wakefield *et al.*, 2015; Wakefield *et al.*, 2016). The snapper were exposed to a 12:12 day/night light cycle and fed *ad libitum* every 24–48 h with teleost fish or squid pieces. Holding tanks were refreshed with a constant flow (50 L min^−1^) of recirculated filtered seawater with a directional manifold that provided a range of circular current velocities for the fish to orient towards. Snapper were housed at 20°C for at least 1 month prior to experiments to allow ample time for metabolic acclimation to occur (Bullock, 1955; Barrionuevo and Fernandes, 1998; Clark *et al.*, 2013).

After respirometry experiments at 20°C, the temperature in the holding tanks was decreased by 1°C d^−1^ to 12°C and fish were transferred into a 5500 L tank with flow-through water at the ambient range edge winter (minimum) temperature of the greater Storm Bay area from which the snapper were collected (Fig. 1). During the cold acclimation period, the hourly mean (± S.D.) temperature was 10.9 ± 0.4°C, with a mean daily range of 1.0 ± 0.6°C. Acclimation temperatures fell within 10–12°C 97% of the time. Experiments were repeated after 10 weeks of acclimation at 12°C. At the time of transfer of each experimental fish from the holding tank to the swim tunnel, the temperature in the holding tank was within 1°C of the treatment temperature (Fig. 1).

**Figure 2:**
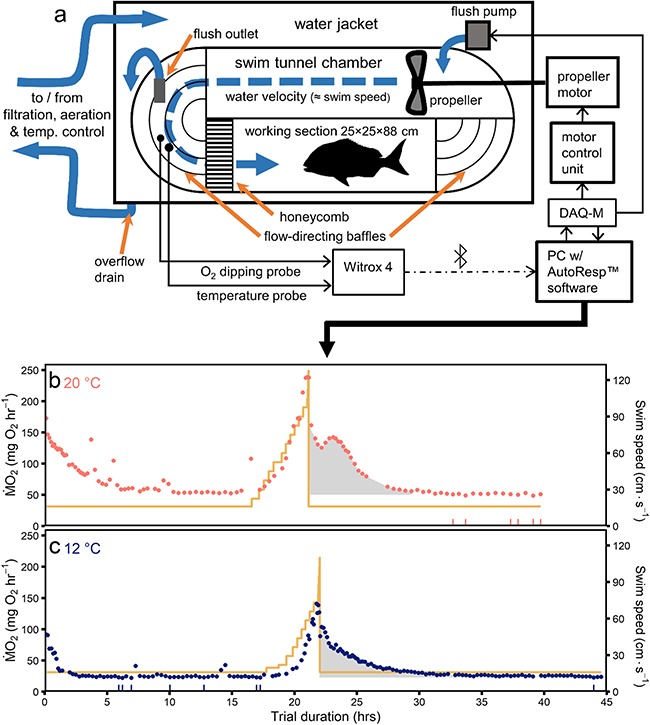
Top-down schematic of the 185 L Loligo swim tunnel respirometry used for snapper swim trials (**a**), and the oxygen consumption (*Ṁ*_O2_) and swimming speed (yellow line) during swim tunnel respirometry trials from a 30 cm, 944 g snapper after acclimation to optimal (**b**) and range edge ambient winter temperature (10–12°C, **c**). The lowest fifth percentile of *Ṁ*_O2_ estimates averaged to estimate standard metabolic rate are indicated by rug tassels on the x axis. EPOC is indicated by the grey area. Note the occasional elevated *Ṁ*_O2_ during the initial acclimation period, typically due to bouts of spontaneous activity, and the decline in *Ṁ*_O2_ after maximum *Ṁ*_O2_ was reached despite increasing swimming speed at 12°C indicating anaerobic metabolism powering swimming.

### Respirometry experiments

Individual snapper were fasted for 48 h prior to respirometry experiments to minimize the influence of post-prandial thermogenesis. Experiments were initiated in the afternoon to allow for acclimation to the respirometer chamber overnight. Each fish was carefully transferred using a silicone net to a padded V-shaped cradle and wet weight (g), total length, width and depth (cm) were recorded. The fish was then placed in a 185 L Loligo® swim tunnel (Fig. 2) with water velocity set to the swim tunnel propeller motor’s minimum (16 cm sec^−1^) or the minimum at which the fish would maintain a steady resting swimming gait if greater. Ambient light was minimized in the room except for a red overhead light and the anterior third of the swim tunnel working section was covered with black PVC fabric to encourage the fish to maintain a steady position in the swim tunnel. Oxygen consumption (*Ṁ*_O2_) was measured as an estimate of aerobic metabolic rate with intermittent respirometry as follows. The swim tunnel was sealed for measurement periods during which a PreSens DP-PSt3 fibre optic oxygen dipping probe recorded the swim tunnel oxygen concentration (*C*_wO2_) every second. *Ṁ*_O2_ was calculated with Loligo AutoResp™ v. 2.2 software as: [(*V*_r_ – *V*_f_) × ΔC_wO2_]∙Δ*t*^-1^, where *V*_r_ and *V*_f_ are the volumes of the respirometer and the fish respectively; Δ*t* is the change in time (*t*) during the measurement period in seconds; and ΔC_wO2_ is the change in oxygen concentration during Δ*t*, calculated as the slope of the linear model ΔC_wO2_ ~ *t* (Clark *et al.*, 2013). The coefficient of determination (R^2^) of the ΔC_wO2_~ *t* model was monitored in real time with AutoResp to estimate optimal Δ*t* to maximize the quantity of reliable *Ṁ*_O2_ estimates and the probability to detect short-term spontaneous changes in *Ṁ*_O2_ (e.g. Fig. 2b, c). The swim tunnel was generally left closed to record *Ṁ*_O2_ until ΔC_wO2_ had fallen to 90% saturation, at which point it was flushed with filtered seawater to replenish oxygen and remove metabolites. Measurements were resumed after a ~90 sec waiting period to allow for C_wO2_ measurements to stabilize after flushing. Fish were closely monitored during the initial acclimation period to ensure water velocity was sufficient for the fish to swim steadily at a resting gait and *Ṁ*_O2_ was stabilizing (Fig. 2).

Once the fish had acclimated to the swim tunnel overnight and *Ṁ*_O2_ estimates stabilized, a swimming trial was initiated (Fig. 1). Water velocity through the swim tunnel chamber was increased in sequential steps by increments proportional to the fish’s body length (BL) to estimate *Ṁ*_O2_ across a range of swimming speeds (*U*_BL_). A correction for solid blocking (the increase in water velocity as it passes the fish due to the fish’s body reducing the effective cross sectional area of the swim tunnel) based on fish dimensions was applied to swimming speed (Bell and Terhune, 1970). The chamber was flushed to replenish oxygen levels as required, typically at the beginning of a swimming speed increment and only *Ṁ*_O2_ estimated from after the first 5 min of each swimming speed increment were considered for analysis.

Maximum sustained swimming speed (*U*_MAX,C_) was determined as the maximum swimming speed reached during which the fish maintained a steady swimming gait without ‘burst’ swimming for the duration of the step (Dickson *et al.*, 2002; McKenzie and Claireaux, 2010). *U*_MAX,C_ is an estimate of the maximum speed an individual can maintain with only or largely aerobic metabolism (Webb, 1971; Rome, 1995; Marras *et al.*, 2013). The switch to burst swimming was determined when the fish could not hold position in the swim tunnel and demonstrated an uneven gait of sliding back in the chamber, followed by swimming forward rapidly in a burst, indicative of the need for anaerobic white muscle to maintain swimming speed. A traditional critical swimming trial (Brett, 1964) was originally planned in swimming speed would continue to increase incrementally until complete exhaustion of the fish. However, in preliminary experiments it was found that snapper have a considerable capacity for burst swimming. The fish would burst swim for multiple 20 min swimming speed increments during which the caudal fin would repeatedly contact the rear grate of the swim tunnel working section in between bursts. In order to prevent damage to the animals’ caudal fins and confounded swimming performance results, a modified design was implemented where at the end of the swimming speed step during which burst swimming began and *U*_MAX,C_ was surpassed, a constant acceleration trial was initiated where speed was increased by 0.25 BL sec^−1^ every 2 min until the fish was exhausted (Fig. 1; Norin and Clark, 2016).

Exhaustion was established as the point at which the fish was in contact with the rear grate of the swim tunnel working section for greater than 3 s more than once in a 30 s period (Dickson *et al.*, 2002). At this point, resting water velocity was resumed, the fish was monitored in the swim tunnel overnight, and *Ṁ*_O2_ was recorded as the fish recovered from the swimming trial until *Ṁ*_O2_ stabilized, at which point the fish was returned to the holding tank. Immediately following experimentation, a blank trial was run for 1–2 h to estimate background respiration in the swim tunnel. The swim tunnel was cleaned with vacuum suction and rinsed in fresh water and occasionally dilute sodium hypochlorite between experiments to inhibit microbial respiration.

### Respirometry analysis

*Ṁ*_O2_ estimates with R^2^ > 0.9 were considered for analysis and were corrected for mean background respiration. Standard metabolic rate (*Ṁ*_O2,MIN_), the minimum metabolic rate required for subsistence, was estimated from the mean of the lowest 5% of *Ṁ*_O2_ during each trial. Maximum metabolic rate (*Ṁ*_O2,MAX_) was determined as the greatest *Ṁ*_O2_ measurement recorded during the trial (Norin and Clark, 2016). Aerobic scope was calculated as both absolute aerobic scope (AS): *Ṁ*_O2_,_MAX_ - *Ṁ*_O2_,_MIN_ and factorial aerobic scope: *Ṁ*_O2,MAX_ ∙ *Ṁ*_O2_,_MIN_^−1^ (Clark *et al.*, 2013; Halsey *et al.*, 2018).

For all metrics involving swimming speed, resting-speed *Ṁ*_O2_ greater than the lowest quintile of all trial *Ṁ*_O2_ were removed to exclude confounding effects of stress or recovery costs. The cost of swimming one body length (or cost of transport, COT) was calculated for each *Ṁ*_O2_ measurement as mass-specific *Ṁ*_O2_ ∙ *U*_BL_^-1^ (mg O_2_ kg^-1^ BL^-1^; Hein and Keirsted, 2011; Videler, 1993). The duration of recovery from exercise was quantified from when the swimming trial was stopped until the first post-exercise *Ṁ*_O2_ equal or less than the lowest quintile of *Ṁ*_O2_. Total mass-specific excess post-exercise oxygen consumption (EPOC) during recovery was quantified as the area under the curve of time versus *Ṁ*_O2_ with trapezoidal Riemann sums. Recovery performance was estimated with the mean proportion of aerobic scope utilized during recovery calculated as (*Ṁ*_O2_ - *Ṁ*_O2,MIN_) ∙ AS^−1^.

**Table 1:** Effects of acclimation temperature on metabolic, swimming and recovery performance metrics. The mean effect of temperature was estimated for mass-specific minimum metabolic rate (*Ṁ*_O2,MIN_), mass-specific maximum metabolic rate (*Ṁ*_O2,MAX_), mass-specific aerobic scope (AS), factorial AS, maximum sustainable swimming speed (*U*_MAX,C_), maximum swimming speed (*U*_MAX_), optimal swimming speed (*U*_OPT_), EPOC duration, total mass-specific EPOC and the mean proportion of aerobic scope used during EPOC with snapper with data at both acclimation temperatures with a mixed effects regression with individual snapper as a random effect. *P*-values were Benjamani–Hochberg corrected for multiple comparisons. Mean individual level Q_10_ temperature coefficients (± 95% confidence interval) are presented for metrics that significantly differed between treatments.

	Acclimation treatment mean ± SD				Effect of treatment 20 °C→12 °C						
					Mean diff.	95% CI		d.f.	p	Q_10_ ± CI
Performance metric	20 °C	n	12 °C	n		Lower	Upper			
Mass (g)	803 ± 441	13	812 ± 428	13	21.7	^−^3.1	46.4	11	0.114	-
*Ṁ*_O2, MIN_ (mg O_2_ kg^−1^ hr^−1^)	111.7 ± 34.2	13	48.3 ± 4.4	13	^−^63.4	^−^82	^−^44.8	11	**<0.001**	2.84 ± 0.52
*Ṁ*_O2, MAX_ (mg O_2_ kg^−1^ hr^−1^)	517.9 ± 54.0	13	293.3 ± 46.1	13	^−^225.5	^−^257.8	^−^193.2	11	**<0.001**	2.08 ± 0.23
AS (mg O_2_ kg^−1^ hr^−1^)	406.2 ± 54.3	13	244.8 ± 46.5	13	^−^162.1	^−^192.6	^−^131.6	11	**0.009**	1.94 ± 0.23
Factorial AS	4.91 ± 1.11	13	6.11 ± 1.08	13	1.20	0.49	1.91	11	**<0.001**	0.78 ± 0.13
*U*_MAX,C_ (body lengths sec^−1^)	2.63 ± 0.43	10	1.91 ± 0.23	11	^−^0.79	^−^1.01	^−^0.57	8	**<0.001**	1.56 ± 0.17
*U*_MAX_ (body lengths sec^-1^)	3.41 ± 0.51	9	2.91 ± 0.36	11	^−^0.44	^−^0.77	^−^0.11	7	**0.042**	1.20 ± 0.15
*U*_OPT_ (body lengths sec^-1^)	1.5 ± 0.39	9	0.81 ± 0.24	13	^−^0.49	^−^0.67	^−^0.32	7	**0.002**	1.70 ± 0.23
EPOC duration (hr)	7.96 ± 2.75	11	12.0 ± 3.83	13	4.40	1.95	6.85	11	**0.008**	0.69 ± 0.24
Total EPOC (mg O_2_ kg^-1^)	715 ± 312	11	463 ± 170	13	^−^254.6	^−^446.6	^−^62.6	11	**0.030**	2.04 ± 0.87
Mean % AS during EPOC	59.4 ± 12.5	11	43.0 ± 6.2	13	^−^16.5	^−^24.1	^−^8.9	11	**0.002**	1.54 ± 0.26

### Statistical analysis

All analyses were conducted with *R* 3.6.0 (R Core Team, 2019). Allometric scaling relationships of fish mass (g) versus *Ṁ*_O2,MAX_ and *Ṁ*_O2_,_MIN_ at each acclimation temperature were characterized with the relationship *Ṁ*_O2_ = *a ∙* mass*^b^*, and the constant *a* and scaling exponent *b* were fit with least squares linear regression of the log-log transformed relationship: log(*Ṁ*_O2_) = *b* ∙ log(mass) + log(*a*) (Clarke and Johnston, 1999). The effects of acclimation temperature and *Ṁ*_O2_ metric (standard versus maximum) on *b* were tested with mixed effects analysis of covariance with interaction terms and with individual as a random effect, fit with the *nlme* package 3.1-140 (Pinheiro *et al.*, 2019). Optimal swimming speed, the speed at which the cost of swimming one body length is minimized, was estimated in each trial as the swimming speed (≤ *U*_MAX,C_) that minimized a weighted third-degree polynomial linear model of the cost of swimming one body length: COT = U_BL_ + U_BL_^2^ + U_BL_^3^. To correct for uneven density of observations across swimming speed (e.g*.* large number of observations at resting speed), each of the *n* total observations at each unique swimming speed value during each trial was weighted in the model by *n*^−1/2^. To compare performance metrics across snapper of different sizes, all metrics were analysed in body length and/or mass-specific forms. Individual-level differences in metrics are presented as Q_10_ temperature coefficients, the amount of change resulting from a temperature increase of 10°C with the formula Q_10_ = (metric_20°C_/metric_12°C_)^10°C/(20°C–12°C)^. To test the effects of acclimation temperature and fish mass on each performance metric, linear mixed effects models were fit with *nlme* to data from fish ran at both acclimation treatments. Models initially included acclimation temperature and mass as fixed effects and individual fish as a random effect. Linear mixed effects models were also used to compare the effects of fish mass, swimming speed and acclimation temperature on the metabolic burden of aerobic swimming with three response metrics: mass-specific *Ṁ*_O2_, mass-specific cost of swimming one body length and the proportion of aerobic scope utilized. The models were constrained to swimming speeds less than or equal to *U*_MAX,C_ and the swimming speed that produced *Ṁ*_O2,MAX_. Above these values, performance metrics are confounded by an increasing anaerobic contribution to swimming performance (Rome, 1995; Rome, 2007; Marras*, et al.*, 2013; Ejbye-Ernst *et al.*, 2016). Swimming speed was modelled as second- and third-degree raw polynomials in the mass-specific *Ṁ*_O2_ and cost of swimming one body length models, respectively. Individual was included as a random effect with correlated random slopes and intercepts. Models were fit with the *lme4* package version 1.1-21 (Bates *et al.*, 2015). Full model details and formulae are available in the supplementary information section, with summaries reported with the *jtools* package version 2.0.1 including pseudo-R^2^ calculated with the Nakagawa and Schielzeth (2013) procedure for linear mixed effects models.

## Results

Swim tunnel respirometry trials were completed with 13 snapper at each acclimation temperature, 12 of which were run at both temperature treatments (an individual that would not swim steadily during 20°C acclimation trials did so at 12°C, another individual that completed a 20°C trial was euthanized prior to 12°C trials due to developing a swim bladder complication). Snapper mass during 20°C trials ranged from 179–1799 g and differed by (mean ± SD) of 4.3 ± 6.1% during subsequent 12°C trials (Table 1). Oxygen consumption (*Ṁ*_O2_) estimates stabilized within ~16 h. Snapper occasionally required a training period of variable water speed of ~5 min before swimming steadily against flow in the swim tunnel. Most fish were found to rest at ~0.5 BL sec^−1^ or less, apart from a few individuals that displayed restless behaviour and variable *Ṁ*_O2_ at the lowest speed and stabilized at ~0.6 BL sec^−1^.

### Allometric scaling of metabolic rate and metabolic performance

**Figure 3:**
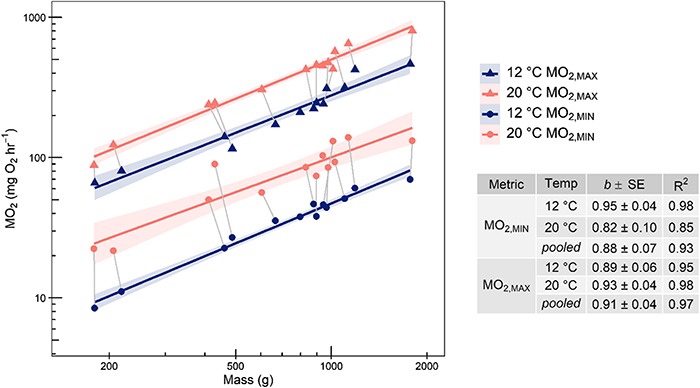
Standard (*Ṁ*_O2,MIN_) and maximum (*Ṁ*_O2,MAX_) metabolic rate estimate allometric scaling relationships by acclimation temperature. Metrics from the same individual during different acclimation trials are connected by grey lines. Estimates of the allometric scaling exponent *b* are presented in the table on the right. Scaling relationships (*Ṁ*_O2_ = *a*∙mass*^b^*) were fit with log-log linear models and scaling exponent *b* pooled across acclimation temperatures were estimated with mixed effects linear models with temperature as a fixed effect and individual as a random effect.

Allometric scaling relationships (*Ṁ*_O2_ = *a* ∙ mass*^b^*) fit with R^2^ of 0.85–0.98, and the pooled estimates for *b* across acclimation temperatures for standard (*Ṁ*_O2,MIN_) and maximum (*Ṁ*_O2,MAX_) metabolic rate were 0.88 and 0.91, respectively (Fig. 3). However, *b* estimates did not significantly vary between metrics (*P* = 0.73) or acclimation temperatures (*P* = 0.54). Mass did not have a significant effect on any of the performance metrics investigated and was subsequently dropped from the effect of acclimation temperature models. Metabolic rate metrics differed significantly between 20°C to 12°C with *Ṁ*_O2,MIN_, *Ṁ*_O2,MAX_ and aerobic scope decreasing while factorial aerobic scope increased (Table 1). The mean individual-level Q_10_ (± 95% C.I., paired *t*-test) of *Ṁ*_O2,MIN_ was 0.75 ± 0.53 greater than the Q_10_ of *Ṁ*_O2,MAX_.

**Figure 4:**
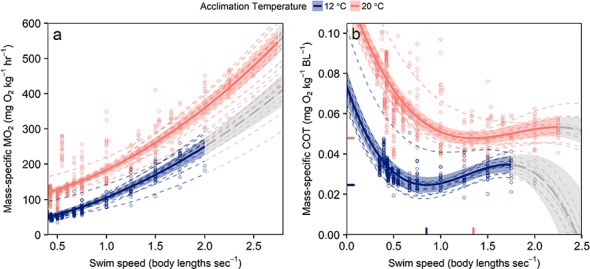
Predicted values of mass-specific oxygen consumption (*Ṁ*_O2_; **a**) and mass-specific cost of transport (COT) (**b**) from linear mixed models with confidence intervals (translucent bands). Dashed lines indicate predicted values at the level of each random effect (individual fish) and points are raw data. Rug tassels on the axes indicate COT minima of the model within the range of sustainable swimming speeds. Note COT declines at high swim speeds presumably due to the contribution of anaerobic metabolism. Predictions beyond the range of sustainable swim speeds at either temperature are represented in grey. Model formulae and coefficient estimates can be found in Table 2 and the supplementary information section.

**Table 2:** Summary table for proportion of swimming performance linear mixed models. The metrics aerobic scope utilized (*%AS*), mass-specific oxygen consumption (*Ṁ*_O2_, mg O2 kg^-1^ hr^-1^) and mass-specific cost of transport (*COT*, mg O2 kg^-1^ body length^-1^) were regressed on predictors acclimation temperature treatment (12°C versus 20°C) and swimming speed (*U*_BL_, body lengths sec^-1^). Beta coefficients are presented with standard error in parentheses. See supplementary information for full model summaries.

	***logit(%AS)***	***Ṁ***_**O2**_	***COT***
(Intercept)	−6.501^***^	25.933^**^	0.074^***^
	(0.159)	(7.103)	(0.004)
Temperature20	1.488^***^	67.592^***^	0.049^***^
	(0.112)	(1.989)	(0.001)
*U*_BL_	4.686^***^	47.265^***^	−0.139^***^
	(0.143)	(11.102)	(0.010)
Temperature20:*U*_BL_	−1.742^***^		
	(0.120)		
*U*_BL_^2^	−6.501^***^	32.347^***^	0.122^***^
		(4.619)	(0.011)
Temperature20:*U*_BL_^2^		10.486^***^	−0.039^***^
		(2.057)	(0.005)
*U*_BL_^3^			−0.032^***^
			(0.004)
Temperature20:*U*_BL_^3^			0.016^***^
			(0.003)
n	1074	1090	1090
σ	0.969	23.102	0.009
R^2^ total	0.845	0.937	0.824
R^2^ fixed	0.790	0.894	0.707
n group (individual)	12	12	12

^***^ *P* < 0.001; ^**^P < 0.01; ^*^P < 0.05.

### Swimming performance

Mass- and body-length-specific maximum sustainable (*U*_MAX,C_), maximum (*U*_MAX_) and optimal swimming speeds all declined with cold acclimation (Table 1). These metrics were calculated for a subset of fish because the swimming speed data from the first two trials were corrupted by interference affecting propeller motor’s analog output signal (subsequently, swimming speed schedules were also recorded with an interference-resistant pencil). The two smallest fish (total length <24 cm) were excluded from *U*_MAX_ comparisons across fish because at high swimming speeds they were observed to take a position in a rear corner of the working section where water velocity was less, confounding swimming speed-performance relationships. At 20°C, *Ṁ*_O2,MAX_ was attained at swimming speeds equal to (36%) or greater than (36%) *U*_MAX,C_ in 72% of trials, while occurring below *U*_MAX,C_ in 27% (χ^2^ = 0.18; df = 2; *P* = 0.91). At 12°C, most (77%) snapper attained *Ṁ*_O2,MAX_ at swimming speeds greater than *U*_MAX,C_, 15% equal to and 8% less than *U*_MAX,C_ (χ^2^ = 11.2; df = 2; *P* = 0.004). In all trials, *Ṁ*_O2_ declined as swimming speed increased after *Ṁ*_O2,MAX_ was reached, despite increasing demands on the fish’s locomotor system.

### Relationship of swimming speed with metabolic rate, cost of transport and aerobic scope utilization

**Figure 5:**
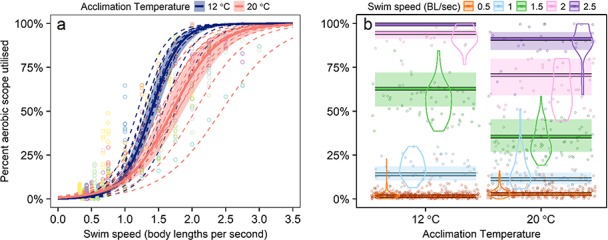
Linear mixed model-predicted proportion (%) of snapper aerobic scope utilized at sustainable swimming speeds with confidence intervals (**a**). Raw data (points) are colour-coded to individual fish and dashed lines are predicted aerobic scope utilization at each random effect level (individual fish). (**b**) Conditional plot of predicted proportion of aerobic scope utilized conditional on swimming speed values, points are partial residuals and violins are kernel-smoothed distributions of partial residuals at each speed. Model formula and coefficient estimates can be found in Table 2 and the supplementary information section.

Fish mass did not have a significant effect on aerobic swimming model fits and was removed. The mean effect of acclimation temperature across the range of swimming speeds resulted in both greater mass-specific *Ṁ*_O2_ and cost of swimming one body length (COT) models at 20°C (Fig. 4, Table 2). There were significant interactions between acclimation temperature and swimming speed in the three aerobic swimming linear mixed models, but with modest effects in the mass-specific *Ṁ*_O2_ and COT models (Table 2, also see supplementary information). The cost of swimming model estimated overall optimal swimming speeds of 0.85 BL s^−1^ at 12°C and 1.35 BL s^−1^ at 20°C (Fig. 4b). The model of proportional aerobic scope utilization demonstrated that cold acclimation allowed low utilization of aerobic scope at low swimming speeds, but at ~0.9 BL sec^−1^ there is an inflection at which the proportional utilization of aerobic scope is consistently greater at 12°C than 20°C (Fig. 5a). At 12°C and 2 BL sec^−1^, over 90% of aerobic scope is required to maintain swimming, while at 20°C the proportion of aerobic scope available is three times greater at the same swimming speed (Fig. 5b).

### Recovery from exercise

*Ṁ*_O2_ declined immediately after the cessation of the swimming trial: the first *Ṁ*_O2_ recorded in the recovery period was 75.0 ± 4.0% (mean ± 95% C.I.) of the last *Ṁ*_O2_ measurement during the swimming trial (one sample *t*-test, t = 8, df = 25, *P* < 0.001; e.g*.*Fig. 2b,c). The greatest *Ṁ*_O2_ during recovery occurred within one to three post-exercise *Ṁ*_O2_ estimates in all trials (<30 min). At 12°C relative to 20°C, total EPOC was less, the duration of EPOC increased despite less total ‘payback’, and the mean proportion of aerobic scope utilized during EPOC decreased (Table 1).

## Discussion

This study presents the first examination of swimming and metabolic performance of a marine fish from the ‘leading edge’ of a climate-driven range extension. We found evidence of performance limitation resulting from range edge minimum temperature acclimation, but support for our three hypotheses was mixed. The hypothesis that aerobic scope would decrease with range edge cold acclimation was supported by absolute, but not factorial aerobic scope. Contrary to the prediction that cold-acclimated performance would decline commensurate with aerobic scope, recovery performance declined despite unused aerobic scope and likewise, sustained swimming failed while aerobic scope was still available. Finally, we hypothesized performances’ proportional utilization of aerobic scope should increase at range edge minimum temperature; however, recovery used proportionally less aerobic scope despite performance decline, and cold-acclimated swimming used proportionally greater aerobic scope at high speed but less at low speeds. Relative to performance at mid-range temperature acclimation, range edge minimum temperature acclimation resulted in a peak swimming performance decline but a swimming efficiency increase. The response of performance to range edge cold acclimation is complex and defies explanation by single metrics. However, these results in conjunction with inferences from elsewhere in the geographic range of snapper and previous work on similar taxa provide insights and suggest a way forward towards understanding cold range limitation.

### Effects of temperature and size on metabolic rate and aerobic scope

**Figure 6:**
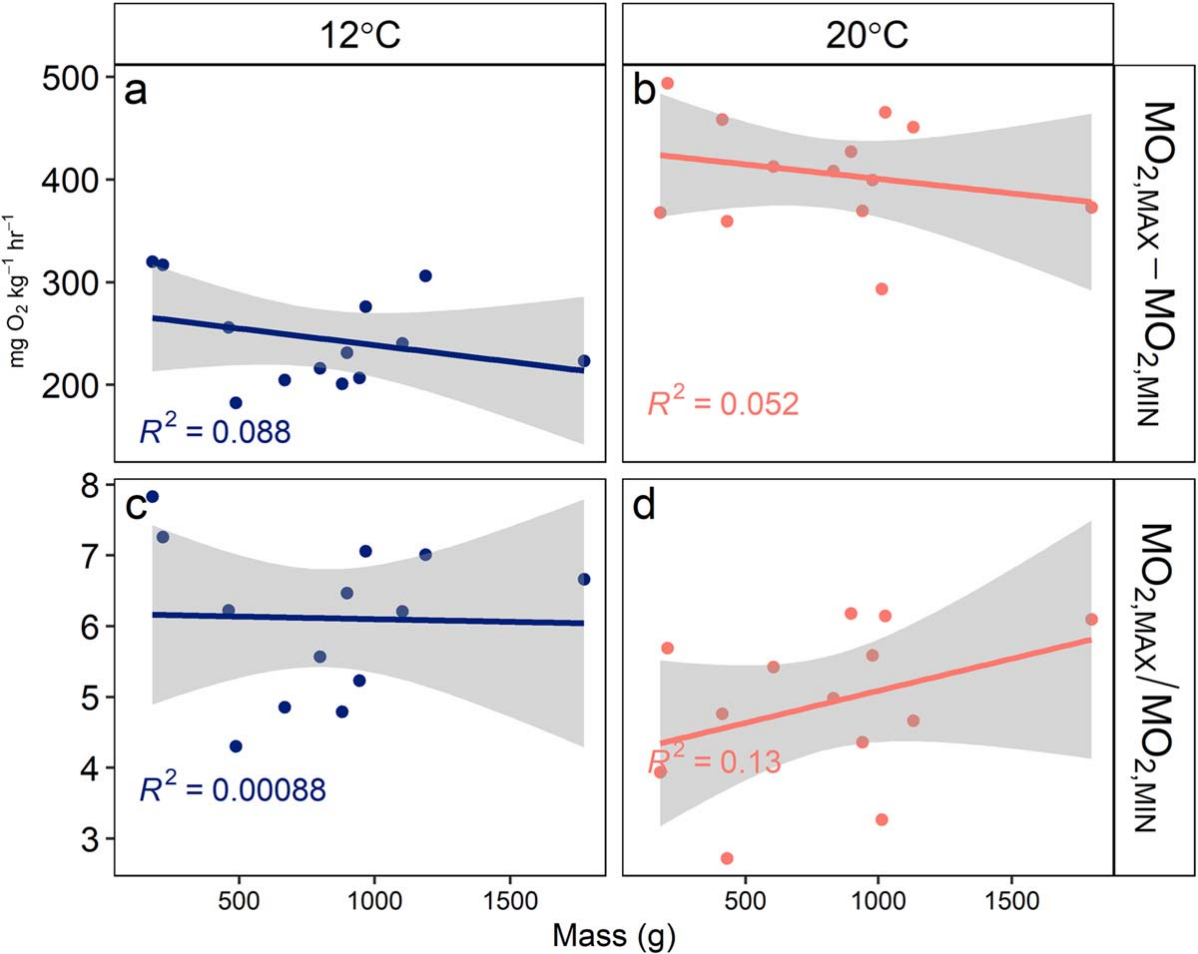
Linear models (with shaded confidence intervals) of individual mass versus aerobic scope by calculation method (mass-specific absolute aerobic scope, **a**, **b**; factorial aerobic scope, **c**, **d**; and acclimation temperature. Each point represents an individual trial.

The 10-fold difference across fish mass in the present study represents late juvenile to adult life stages (Fig. 3). Standard metabolic rate (*Ṁ*_O2,MIN_) increased with a greater allometric scaling exponent *b* than a number of teleost species reported (mean = 0.79, Clarke and Johnston, 1999) but was consistent with *b* of other subcarangiform swimmers (Killen *et al.*, 2007). The *b* of maximum metabolic rate (*Ṁ*_O2,MAX_) in the present study were not significantly greater than those of *Ṁ*_O2,MIN_; however, the interaction between metric and acclimation temperature approached significance with this sample size (*P* = 0.13). This contrasts with prior theoretical predictions and evidence from other teleost species that *Ṁ*_O2,MAX_ increases more sharply with mass than *Ṁ*_O2,MIN_ as a result of volume-related muscle power production (Glazier, 2005; Killen *et al.*, 2007; Glazier, 2009). It has been suggested that greater *b* may have fitness implications for smaller individuals because factorial aerobic scope increases with size when *Ṁ*_O2,MAX_ scales with a greater *b* than that of *Ṁ*_O2,MIN_ (Killen *et al.*, 2007). The allometric scaling of metabolism has implications across ontogenetic stages in range extensions if at mid-range but not range edges, fish benefit from *Ṁ*_O2,MAX_ increasing faster with mass than *Ṁ*_O2,MIN_. These results suggest that while this trend may be present at 20°C and not 12°C (Fig. 6b), other sources of intraspecific variation likely buffer the implications of allometric trends in metabolic performance as the relationships between mass and both aerobic scope metrics were weak (R^2^ = 0 – 0.13; Fig. 6).

In some teleost species, with sufficient acclimation time (4–8 weeks) after introduction to above-optimal temperatures, near-complete compensation of *Ṁ*_O2,MIN_ can occur (Grans *et al.*, 2014; Sandblom *et al.*, 2014). These examples are of acclimation to greater than optimal temperature in which compensation decreases *Ṁ*_O2,MIN_ likely to preserve aerobic scope. However, very limited work has been done on the effects of long term fish acclimation on aerobic scope, especially to below-optimal temperatures (Clark *et al.*, 2013). At cold temperatures, the uncompensated decline in *Ṁ*_O2,MIN_ due to Q_10_ may be a benefit to the degree that it reduces ‘maintenance costs’ if the capacity for activity is preserved. In the present study, the individual-level effect of 10 weeks of cold acclimation on *Ṁ*_O2,MIN_ (Q_10_ = 2.84 ± 0.52) is approximately equal to the median intraspecific Q_10_ = 2.4 calculated across 14 species by Clarke and Johnston (1999), while the significantly smaller Q_10_ of *Ṁ*_O2,MAX_ (2.08 ± 0.23) suggests oxygen transport capacity is conserved at low temperatures. Considerable variation in individual-level Q_10_ of metabolic rate metrics (Table 1) likely reflects population variation of metabolic phenotypes (Duncan *et al.*, 2019).

The dichotomy between the effects of cold acclimation on snapper aerobic scope and factorial aerobic scope adds to a number of studies that have reached conflicting conclusions on the effect of temperature depending on whether aerobic scope is considered as a proportion or an absolute amount (Clark *et al.*, 2011; Donelson *et al.*, 2012). Is the 29% mean increase in factorial aerobic scope or the 39% mean decrease in absolute aerobic scope the relevant ‘capacity’ metric when considering potential for success at range edges? For practical purposes, absolute aerobic scope is favourable because it is less prone to *Ṁ*_O2,MIN_ estimation errors (Clark *et al.*, 2013; Halsey *et al.*, 2018). However, if the energetic costs of fitness-related performances like digestion, locomotion and growth scale with temperature, factorial aerobic scope may be more informative than absolute changes in capacity. For example, if cold exposure reduces absolute aerobic scope but also reduces performance costs at a greater rate, a calculated decline in aerobic capacity would result in a paradoxically greater free proportion of available capacity. Energy allocation modelling of cod predicts that proportional utilization of aerobic scope would be greatest at high and low temperatures, suggesting factorial aerobic scope is pertinent (Holt and Jørgensen, 2015). However, reduced performance may be due to factors independent of physiological limitation, e.g. an adaptive behavioural strategy (Careau *et al.*, 2008; Biro *et al.*, 2018). Growth of wild snapper across their first 3 years of life virtually halts when temperatures fall below 14°C suggesting limitation or a trade-off involving somatic growth (Francis, 1994). The effects of temperature on the energetic costs and performance of other fitness-linked traits such as digestive, reproductive or immune capacity is an important area for future work.

### Swimming performance and efficiency

Despite their widespread abundance and importance to fisheries across Australia and New Zealand, this is the first known study of snapper exercise respirometry beyond the juvenile life stage (~200 g). Snapper are good candidates for swim tunnel experimentation, requiring little to no training period before stable swimming. In one of the few prior studies of snapper metabolic rate, Cook and Herbert (2012) estimated ~225 g snapper *Ṁ*_O2,MIN_ = 136.6 ± 12.7 mg O_2_ kg^−1^ hr^−1^ at 18°C in a 5L static respirometer, which compares to the current study’s 101.4 ± 15.1 mg O_2_ kg^−1^ hr^−1^ after adjusting for Q_10_ and estimation method. The discrepancy may be due in part to the swim tunnel method allowing for some *Ṁ*_O2_ estimation at lower spontaneous activity or stress levels. However, snapper in the present study did appear to be sensitive to insufficient water velocity. At low water velocities, snapper did not rest on the bottom of the swim tunnel and relied on fin propulsion to maintain position in the swim tunnel. Some individuals’ *Ṁ*_O2_ became unsteady and increased as the fish were observed to use median and paired fin propulsion for positioning until swimming speed was increased and the fish switched over to steady caudal propulsion. While using median and paired fins at insufficient water velocity for steady caudal propulsion, *Ṁ*_O2_ was similar to that of moderate swimming speeds (≈1.5 BL s^−1^). The subcarangiform gait is presumably more efficient than use of median and paired fins for locomotion at moderately low swimming speed but not useful below 0.5 BL s^−1^. These resting speeds are similar to the varying spontaneous swimming speeds of juvenile snapper (~20 cm) in the absence of directed water velocity (0.44 ± 0.08 BL s^−1^_,_Cook *et al*., 2011; 0.53 ± 0.07 BL s^−1^ and 0.65 ± 0.10 BL s^−1^, Cook and Herbert, 2012). These results suggest snapper may prefer areas with either negligible or moderate water flow at rest to minimize the cost of maintaining position; however, energetic inefficiency around the gait transition speed are likely exacerbated by confinement in the swim tunnel as it prevented turning.

At 20°C, the mean maximum sustainable swimming speed (*U*_MAX,C_) of 2.63 ± 0.43 BL s^−1^ was lower than measured in other teleosts with a similar swimming gait. Saithe *Pollachius viriens* (25.5 cm) maintained 3.84 BL s^−1^ for 30 min, 25.3 cm herring *Culpea harengus* maintained 4.8 BL s^−1^ for 40 min and 26 cm green jack *Caranx caballus* 4.5 BL s^−1^ (Dickson *et al.*, 2002). Differences may be due to methodological differences, the larger relative size of snapper or adaptation to different ecological niches. The decline of *U*_MAX,C_ in snapper with decreasing temperature is consistent with previous findings and is likely due to lower power output of slow oxidative (red) muscle at cold temperature (Brett and Glass, 1973; Taylor *et al.*, 1996; Dickson *et al.*, 2002). As drag is nearly temperature independent (Q_10_ ≈ 1.1), virtually the same power is required to power swimming across acclimation temperatures (Rome, 2007). At 12°C, the red muscle is likely unable to produce the amount of power at *U*_MAX,C_ as at 20°C, and fast glycolytic (white) muscle must be recruited to power the same swimming speed (Rome and Sosnicki, 1990; Rome *et al.*, 1992; Taylor *et al.*, 1996; Dickson *et al.*, 2002; Rome, 2007).

In the well-studied scup *Stenotomus crysops*, another sparid with a similar body plan and life history as snapper, pink muscle is recruited at *U*_MAX,C_. Pink muscle is positioned between red and white muscle and likewise intermediate in substrate use (i.e. less aerobic than red). It is critical for supporting scup swimming performance in the cold, providing 18 times the amount of power per unit mass in 10°C at 50 cm s^−1^ compared 20°C but only 1.5 times the power per unit mass at 80 cm s^−1^ (Rome, 2007). The increasing contribution of pink muscle to swimming as temperatures fall below optimal demonstrates the dynamic role of non-aerobic processes in supporting routine activity across the thermal window. Sustainable swimming at low temperature is highly dependent on acclimation time, in part due to non-structural changes during acclimation that are independent of aerobic metabolism. Despite the strong negative effect of cold on muscle shortening velocity (Hill, 1964), the warm-acclimated (20°C) scup nervous system does not allow for a longer relaxation period between stimulations of red muscle in cold (10°C) conditions and thus their aerobic muscles are inefficient, doing little or even negative work (Rome, 2007). However, the inverse is not the case, and cold acclimated scup use efficient enervation to optimize muscle performance in warm water. Cold acclimation can also affect swimming through structural changes like red muscle hypertrophy, for example ~50% more red muscle mass in the striped bass *Morone saxatilis* (Jones and Sidell, 1982).

Most of the 12°C acclimated snapper reached full aerobic scope utilization (*Ṁ*_O2,MAX_) after anaerobic burst swimming had begun (i.e. above *U*_MAX,C_). The failure of aerobic swimming while aerobic scope was available suggests the cold-induced decline in aerobic swimming was not due to aerobic scope limitation but at least partially due to failure of muscle performance, which can arise with cold at the tissue/cellular level (MacMillan, 2019). These results suggest cold-acclimated snapper are optimized for sedentary behaviour: low optimal swimming speed and low utilization of aerobic scope at swimming speeds less than 1 BL s^−1^, poor mid-range aerobic swimming, but a conserved capacity to burst swim.

### Recovery from exhaustive exercise

While payback of the oxygen debt incurred during exercise (EPOC) at 12°C required a longer duration despite relatively less total debt, aerobic scope limitation was not apparent here as cold-acclimated fish utilized less of their available aerobic scope during recovery than at 20°C, when a greater proportion of aerobic scope was used to pay back a greater debt in a shorter period. It has been suggested that *Ṁ*_O2,MAX_ reached while ram ventilating at high swimming speed may be unreachable at slow speeds due to limits on gill perfusion (Clark *et al.*, 2013). This would inflate resting aerobic scope estimates (and underestimate proportional aerobic scope utilization) during recovery. Thus, the relative decline of the cold-acclimated proportion of aerobic scope during EPOC may be even greater, as *Ṁ*_O2,MAX_ at 20°C was estimated at greater swimming speed than at 12°C with greater potential gill perfusion. A related implication of these results concerns methodology in assessing fish metabolism. Maximum metabolic rate is often estimated in a static respirometer chamber of an animal after the cessation of an exhaustive manual chase protocol (Clark *et al.*, 2013; Norin and Clark, 2016). The peak *Ṁ*_O2_ during recovery or even *Ṁ*_O2,MAX_ occurs hours after returning to rest for some fishes (Bushnell *et al.*, 1994). In contrast, during the present study *Ṁ*_O2,MAX_ was sometimes reached while swimming at near-maximum speed and *Ṁ*_O2_ declined considerably immediately after exercise ceased. Therefore, a chase-and-measure protocol likely would not produce an *Ṁ*_O2,MAX_ estimate comparable to one recorded during a swim tunnel trial. Care should be taken when comparing estimates of *Ṁ*_O2,MAX_ of snapper and similar taxa from different methodologies.

### Importance and limitations of studying performance at the range extension front

As range extensions are complex phenomena resulting from processes interacting across multiple biological and environmental scales (Bates *et al.*, 2014), to understand underlying mechanisms it is necessary to quantify these processes or otherwise account for them. The segment of the population at the range extension front may vary from the population means across physiological and behavioural traits (e.g. ‘boldness’, Myles-Gonzalez *et al.*, 2015). Range edge animals may have a subset of phenotypic variations of the larger population and different long-term acclimation temperature histories. For example, snapper vary in the size at maturity by over 2-fold across their range, and the size and age at maturity increases with latitude (Wakefield *et al*., 2016). Collecting experimental animals at a range extension front provides a simple way to control for any relative intraspecific performance differences that may occur in range extending individuals to more accurately identify mechanisms active at range extensions. Expanding range extension front data into a macrophysiological approach (with samples across large geographical scales, Chown and Gaston, 2008) would allow for quantification of physiological variation across species ranges and its implications for species redistributions to be investigated (Present and Conover, 1992; Fangue *et al.*, 2006; Osovitz and Hofmann, 2007).

Research on the distributional extremes of a population presents several logistical challenges. The ongoing range extension must first be identified. Animals are typically difficult to obtain due to low abundances and limited ecological information, and experimental equipment often is not in proximity. To obtain experimental fish in the present study, the recreational fishing community was closely consulted as catching snapper even sporadically in southeast Tasmania requires very specific targeting. With limited animal availability, instead of attempting to control for the effect of size, we instead used it as a feature of the study to investigate allometric relationships, and with a repeated measures design to control for individual-level variance, found no significant effect of mass on performance metrics compared across acclimation temperature.

### Applications and future directions

While many assumptions about what traits are fitness-related or limiting at sub-optimal temperatures are probably reasonable, especially towards critical thermal limits, some are perhaps less grounded. For example, what are the fitness implications of the high relative costs of snapper swimming at moderate speeds at cold temperatures? While it is commonly assumed animals have evolved so that optimal swimming speed coincides with typical cruising speed at range-optimal temperatures, this does not account for trade-offs required to maintain acclimation capacity across the seasonal window nor trade-offs with other fitness-conferring traits (Jørgensen *et al.*, 2016). Priede (1985) suggested efficiency has no necessary link with fitness, as poor efficiency is acceptable if it ultimately allows for a gain in reproduction. These important gaps could be addressed with field data of snapper. While cold-acclimated swimming above low speeds used more proportional aerobic scope as predicted, use of swimming speeds across temperature by snapper *in situ* is required to determine whether this results in actual energy budget conflicts as suggested by modelling (Holt and Jørgensen, 2015). Additionally, understanding the fine-scale thermal habitat availability and use of snapper would provide further context, as utilization varies spatially and fishes are known to use behavioural thermoregulation to moderate thermal conditions experienced (Hight and Lowe, 2007; Ward *et al.*, 2010; Campana *et al.*, 2011).

The present study has examined the performance of juvenile and subsequent life stages at which snapper are known to be capable of long migrations (Hamer and Jenkins, 2004; Harasti *et al.*, 2015), suggesting these life stages could be driving the range expansion. However, anecdotal reports of very small juveniles occurring in southeast Tasmania suggest larval recruitment may have a role. The relative degree to which larval transport and poleward migration of older snapper contribute to the range-extending population is an important knowledge gap, as a redistribution driven by the former is likely temporally and spatially variable (Potts *et al.*, 2014).

Many temperate fish are able to over-winter in a sedentary state that results from the physicochemical effects (i.e. Q_10_) of cold on *Ṁ*_O2_ and reduced activity rates for energy savings (Speers-Roesch *et al.*, 2018). For these taxa, acute periods of sub-lethal low temperature alone are unlikely to result in range-limiting fitness impacts. Other areas associated with high snapper abundances reach the cold treatment acclimation temperature of the present study (e.g. Port Phillip Bay, Victoria, annual minimum weekly mean water temperature ≈ 10°C). Despite similar minimum temperatures at the range edge 550 km to the south, Port Phillip Bay is productive snapper habitat and a source of recruitment for the snapper population across 1000 km of coastline (Hamer *et al.*, 2011). The disparity is likely due to higher mean annual temperatures in Port Phillip Bay. Cold exposure is likely to affect fitness based on length of time at dormancy-promoting temperature versus those that favour increased energy throughput. Recent work on freshwater fishes demonstrates energy acquisition during warm months is critical for fitness and overwinter survival (Fernandes and McMeans, 2019).

As a way forward to understand cold range limitation, we suggest wherever possible moving beyond the use of theoretical ‘master key’ metrics or attempting to isolate single responsible performances. Identifying performance metrics that collapse at extreme temperatures rather than sub-critical effects at ecologically relevant range edge temperatures may not be informative. Considering these results from the range edge along with the success of snapper in areas that reach equally low minimum but higher maximum temperatures, range limitation likely results from cumulative time exposed to suboptimal temperature. As individual performances are linked to survival and fitness through their influence on energy throughput (Brown, 1995), we suggest employing a bioenergetic modelling approach (Jørgensen *et al.*, 2016; Speers-Roesch and Norin, 2016) towards understanding range limitation. Incorporating physiological performance, *in situ* seasonal temperature exposure and ecological data into models of range limitation will improve the resolution and accuracy of forecasts over both space and time. By ground truthing model predictions against observations from this well-studied species across its range (e.g. abundance, growth and reproductive rates), the contribution of specific mechanisms underpinning performance limitation and trade-offs between them can be assessed to guide future empirical work and develop forecasts of range dynamics.

## Funding

This work was supported by the Australian Research Council [FT140100596 to G.P.]

## Supplementary Material

Supp_Info-Wolfe_Cold_Snapper_rev
