# Supplementary material for: Physiological mechanisms linking cold acclimation and the poleward distribution limit of a range-extending marine fish
Source: Conserv Physiol. 2020 Sep 8;8(1):coaa045. doi: 10.1093/conphys/coaa045 (PMC7248536; doi:10.1093/conphys/coaa045)
Supplement: Supp_Info-Wolfe_Cold_Snapper_rev [file supp_info-wolfe_cold_snapper_rev.docx]

**Physiological mechanisms linking cold acclimation and the poleward distribution limit of a range-extending marine fish**

Barrett W. Wolfe, Quinn P. Fitzgibbon, Jayson M. Semmens, Sean R. Tracey, Gretta T. Pecl

**Supplementary information**

This file contains :

- Model summaries for aerobic swimming linear mixed models in the Results subsection *Relationship of swimming speed with metabolic rate, cost of transport and aerobic scope utilisation*; Table 2 and Figures 4 & 5.
- Contour plots and model summaries for the models ran with acclimation temperature treated as a continuous variable to allow interpolation between treatment temperatures.

**Model Summary:** Swimming speed (*U*_BL_, body lengths sec^-1^) & acclimation temperature vs mass-specific oxygen consumption (*Ṁ*_O2_, mg O2 kg^-1^ hr^-1^)

| **Observations** | | 1090 | | | | | | | | | | | | | |  |  |
| --- | --- | --- | --- | --- | --- | --- | --- | --- | --- | --- | --- | --- | --- | --- | --- | --- | --- |
| **Model** | | *Ṁ*_O2_ **~** *U*_BL_ + *U*_BL_^2^ × Temperature + (*U*_BL_ \| Individual) | | | | | | | | | | | | | |  |  |
| **Type** | | Mixed effects linear regression | | | | | | | | | | | | | |  |  |
| **Pseudo-R² (fixed effects only)** | | | | | | | | | | 0.89 | | | | |  |  |  |
| **Pseudo-R² (fixed & random effects)** | | | | | | | | | | 0.94 | | | | |  |  |  |
| **Fixed Effects** | | | | | | | | | | | | | | | | | |
|  | | **Est.** | | **2.5%** | | | | | **97.5%** | | | | **t val.** | **d.f.** | | | **p** |
| (Intercept) | | 25.9 | | 11.83 | | | | | 40.2 | | | | 3.65 | 16.9 | | | 1.99e-03 |
| *U*_BL_ | | 32.3 | | 23.18 | | | | | 41.3 | | | | 7.00 | 1067.5 | | | 4.41e-12 |
| Temp20 | | 67.6 | | 63.70 | | | | | 71.5 | | | | 33.99 | 1075.2 | | | 1.47e-172 |
| *U*_BL_^2^ | | 47.3 | | 25.51 | | | | | 68.9 | | | | 4.26 | 60.9 | | | 7.26e-05 |
| Temp20:*U*_BL_^2^ | | 10.5 | | 6.47 | | | | | 14.5 | | | | 5.10 | 1041.6 | | | 4.07e-07 |
| p values calculated using Satterthwaite d.f. | | | | | | | | | | |  |  |  |  |  |  |  |
| **Random Effects** | | | | | | | | | | |  |  |  |  |  |  |  |
| **Group** | | **Parameter** | | | | | | **Std. Dev.** | | | |  |  |  |  |  |  |
| Individual | | (Intercept) | | | | | | 21.5 | | |  |  |  |  |  |  |  |
| Individual | | *U*_BL_ | | | | | | 23.9 | | |  |  |  |  |  |  |  |
| Residual | |  | | | | | | 23.1 | | |  |  |  |  |  |  |  |
| **Grouping Variables** | | | | |  | |  |  |  |  |  |  |  |  |  |  |  |
| **Group** | **# groups** | | **ICC** | | |  |  |  |  |  |  |  |  |  |  |  |  |
| Individual | 12 | | 0.46 | | |  |  |  |  |  |  |  |  |  |  |  |  |

**Model Summary:** Swimming speed (*U*_BL_, body lengths sec^-1^) & acclimation temperature vs mass- and body-length-specific cost of transport (COT, mg O2 kg^-1^ body length^-1^)

| **Observations** | 1090 | | | | | | | | | |  |
| --- | --- | --- | --- | --- | --- | --- | --- | --- | --- | --- | --- |
| **Model** | COT ~ *U*_BL_ + (*U*_BL_^2^ + *U*_BL_^3^) × Temperature + (*U*_BL_ \| Individual) | | | | | | | | | |  |
| **Type** | Mixed effects linear regression | | | | | | | | | |  |
| **Pseudo-R² (fixed effects only)** | | | | | | 0.71 | | |  |  |  |
| **Pseudo-R² (fixed & random effects)** | | | | | | 0.82 | | |  |  |  |
| **Fixed Effects** | | | | | | | | | | | |
|  | **Est.** | **2.5%** | | | **97.5%** | | **t val.** | **d.f.** | | | **p** |
| (Intercept) | 0.0738 | 0.0656 | | | 0.0821 | | 17.59 | 34.4 | | | 8.97e-19 |
| *U*_BL_ | -0.1386 | -0.1584 | | | -0.1186 | | -13.62 | 943.1 | | | 1.11e-38 |
| Temp20 | 0.0486 | 0.0463 | | | 0.0510 | | 40.69 | 1073.1 | | | 1.09e-219 |
| *U*_BL_^2^ | 0.1219 | 0.1001 | | | 0.1436 | | 10.96 | 1042.8 | | | 1.63e-26 |
| *U*_BL_^3^ | -0.0317 | -0.0394 | | | -0.0241 | | -8.10 | 1025.3 | | | 1.56e-15 |
| Temp20:*U*_BL_^2^ | -0.0394 | -0.0486 | | | -0.0303 | | -8.42 | 1063.1 | | | 1.19e-16 |
| Temp20:*U*_BL_^3^ | 0.0164 | 0.0112 | | | 0.0215 | | 6.20 | 1064.1 | | | 7.83e-10 |
| p values calculated using Satterthwaite d.f. | | | | | | | | | |  |  |
| **Random Effects** | | | | | | | | | |  |  |
| **Group** | **Parameter** | | | **Std. Dev.** | | | | | |  |  |
| Individual | (Intercept) | | | 0.010 | | | | | |  |  |
| Individual | *U*_BL_ | | | 0.006 | | | | | |  |  |
| Residual |  | | | 0.009 | | | | | |  |  |
| **Grouping Variables** | | | | | |  |  |  |  |  |  |
| **Group** | **# groups** | | **ICC** | | |  |  |  |  |  |  |
| Individual | 12 | | 0.57 | | |  |  |  |  |  |  |

**Model Summary:** Swimming speed (*U*_BL_, body lengths sec^-1^) & temperature vs percent aerobic scope (%AS) utilised

| **Observations** | | | 1074 | | | | | | | | | |  |  |
| --- | --- | --- | --- | --- | --- | --- | --- | --- | --- | --- | --- | --- | --- | --- |
| **Model**^†^ | | | logit(%AS) ~ *U*_BL_ * Temperature + (*U*_BL_ \| Individual) | | | | | | | | | |  |  |
| **Type** | | | Mixed effects linear regression | | | | | | | | | |  |  |
| **Pseudo-R² (fixed effects only)** | | | | | | | | | 0.79 | |  |  |  |  |
| **Pseudo-R² (fixed & random effects)** | | | | | | | | | 0.84 | |  |  |  |  |
| **Fixed Effects** (logit scale) | | | | | | | | | | | | | | |
|  | | **Est.** | | | **2.5%** | | | **97.5%** | | **t val.** | | **d.f.** | | **p** |
| (Intercept) | | -6.50 | | | -6.82 | | | -6.18 | | -40.82 | | 13.84 | | 7.98e-16 |
| Temp20 | | 1.49 | | | 1.27 | | | 1.71 | | 13.23 | | 868.50 | | 1.52e-36 |
| *U*_BL_ | | 4.69 | | | 4.40 | | | 4.97 | | 32.75 | | 19.91 | | 8.67e-19 |
| Temp20:*U*_BL_ | | -1.74 | | | -1.98 | | | -1.51 | | -14.55 | | 709.31 | | 3.65e-42 |
| p values calculated using Satterthwaite d.f. | | | | | | | | | | | | | | |
| **Random Effects** | | | | | | |  |  |  |  |  |  |  |  |
| **Group** | **Parameter** | | | **Std. Dev.** | | |  |  |  |  |  |  |  |  |
| Tag | (Intercept) | | | 0.485 | | |  |  |  |  |  |  |  |  |
| Tag | *U*bl | | | 0.380 | | |  |  |  |  |  |  |  |  |
| Residual |  | | | 0.969 | | |  |  |  |  |  |  |  |  |
| **Grouping Variables** | | | | | |  |  |  |  |  |  |  |  |  |
| **Group** | **# groups** | | | **ICC** | |  |  |  |  |  |  |  |  |  |
| Individual | 12 | | | 0.20 | |  |  |  |  |  |  |  |  |  |
|  |  | | |  | |  |  |  |  |  |  |  |  |  |

† %AS approached 0 and 1 slowly (∝ *Ṁ*_O2_ near *Ṁ*_O2,Min_ and *Ṁ*_O2,Max_ respectively), thus the log-odds of %AS was normally distributed. To fit model assumptions the metric was first scaled by reducing values of 1 by 0.0025 and excluding values ≤ 0 to fit the bounds of (0,1), and then logit (log(x/(1-x)) transformed.

**Table S1.** Summary tables for linear mixed models with temperature treated as a continuous variable. Model formulae are the same as in Table 2 and the summaries in this supplement, with the exception of the mass-specific metabolic rate model which is also provided without an interaction term between the predictors swim speed (*U*_BL_) and temperature.

|  | *%AS* | *ṀO2* | *ṀO2 (no int)* | *COT* |
| --- | --- | --- | --- | --- |
| (Intercept) | -8.733 *** | -75.455 *** | -78.207 *** | 0.001 |
|  | (0.266) | (7.610) | (7.722) | (0.004) |
| Temperature | 0.186 *** | 8.449 *** | 9.183 *** | 0.006 *** |
|  | (0.014) | (0.249) | (0.205) | (0.000) |
| *U*_BL_ | 7.299 *** | 47.265 *** | 23.904 * | -0.139 *** |
|  | (0.280) | (11.102) | (10.410) | (0.010) |
| Temperature : *U*_BL_ | -0.218 *** |  |  |  |
|  | (0.015) |  |  |  |
| *U*_BL_ ^2^ |  | 16.618 * | 49.572 *** | 0.181 *** |
|  |  | (7.187) | (3.193) | (0.017) |
| Temperature : *U*_BL_ ^2^ |  | 1.311 *** |  | -0.005 *** |
|  |  | (0.257) |  | (0.001) |
| *U*_BL_ ^3^ |  |  |  | -0.056 *** |
|  |  |  |  | (0.008) |
| Temperature : *U*_BL_ ^3^ |  |  |  | 0.002 *** |
|  |  |  |  | (0.000) |
| n | 1074 | 1090 | 1090 | 1090 |
| sigma | 0.969 | 23.102 | 23.358 | 0.009 |
| logLik | -1525.499 | -4999.851 | -5012.250 | 3479.591 |
| deviance | 3032.356 | 10012.171 | 10038.012 | -7051.248 |
| df.residual | 1066 | 1081 | 1082.000 | 1079 |
| R^2^ total | 0.845 | 0.937 | 0.935 | 0.824 |
| R^2^ fixed | 0.790 | 0.894 | 0.888 | 0.707 |
| n group (individual) | 12 | 12 | 12 | 12 |
|  | *** p < 0.001; ** p < 0.01; * p < 0.05. | | | |


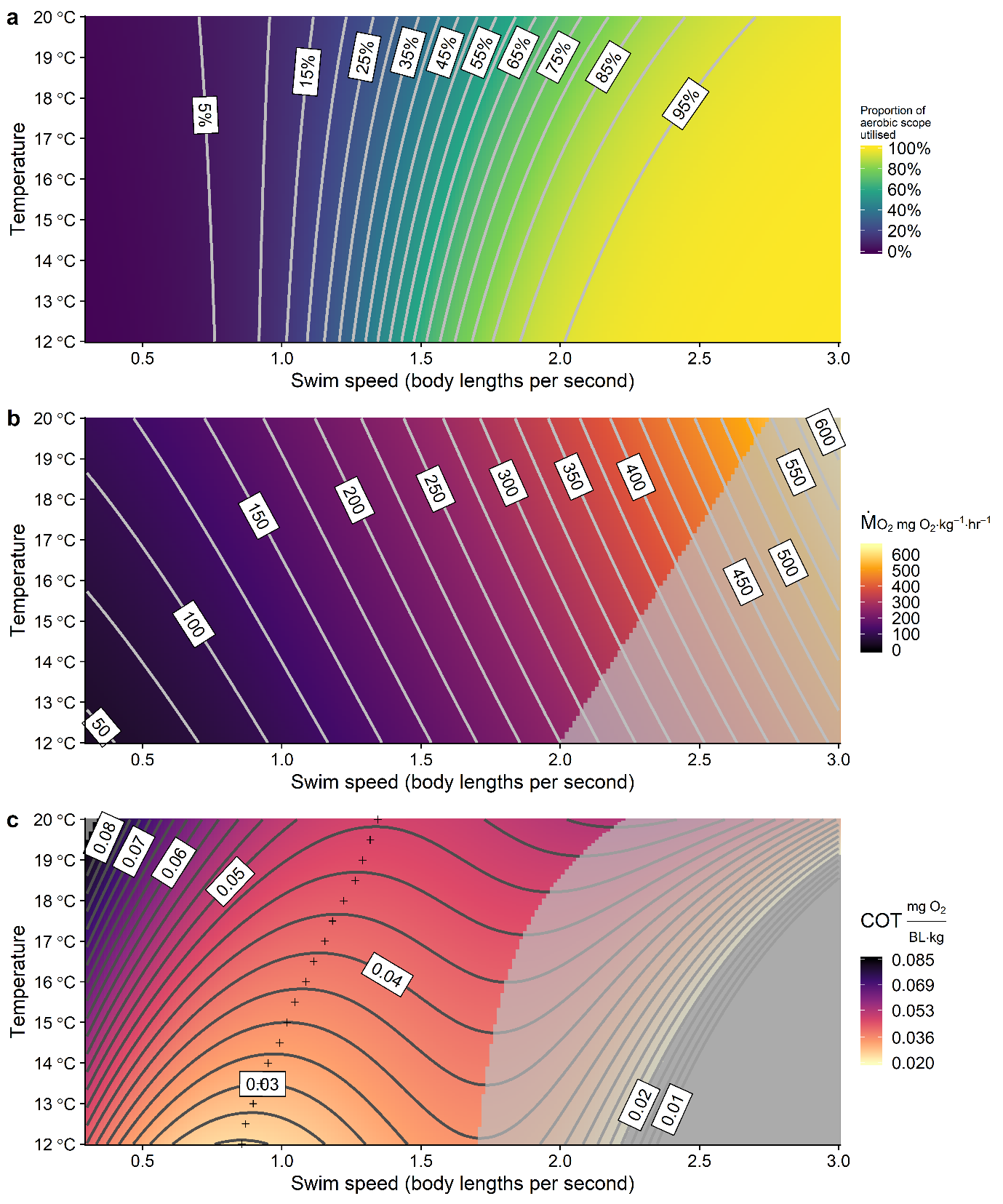


**Figure S1.** Contour plots of linear mixed models of: proportion of aerobic scope utilised, **a**; mass-specific oxygen consumption (*Ṁ*_O2_), **b**; and the cost of swimming one body length (COT), **c**; vs temperature and swimming speed. Outputs are from the same models displayed in figures 4 and 5 in the main text with the change that temperature is treated as a continuous variable instead of two factors (12 °C and 20 °C). Greyed areas in the *Ṁ*_O2_ model plot (**b**) indicate values above the range of sustainable swimming speed to which the *Ṁ*_O2_ model was fit, and in the COT model plot (**c**) indicate values where COT declines with increasing swim speed as an artefact (assumed due to an increasing contribution of anaerobic metabolism powering swimming). The ‘+’ symbols in **c** indicate the optimal swimming speed at each half-degree Celsius increment (calculated as the minima of COT within the range of sustainable swimming speeds.
